# Supplementary material for: Water-Soluble Truncated Fatty Acid–Porphyrin Conjugates Provide Photo-Sensitizer Activity for Photodynamic Therapy in Malignant Mesothelioma
Source: Cancers (Basel). 2022 Nov 5;14(21):5446. doi: 10.3390/cancers14215446 (PMC9654571; doi:10.3390/cancers14215446)
Supplement: Supplementary file 1 [file cancers-14-05446-s001.zip › cancers-1866149-supplementary.pdf]

Supporting Information

5,10,15,20-Tetrakis(ethyl-5-(3-phenoxy)pentanoate)-21H,23H-porphyrin, 2

To 1 (0.53g, 0.78 mmol) and Cs_2CO_3 (1.22g, 3.75 mM) mmol was added acetonitrile (50ml). The reaction mixture was stirred at reflux for 15 min protected by an N_2 atmosphere. To this reaction mixture ethyl-5-bromovalerate (0.78g, 3.75 mmol) was added and refluxed for a further 41 hrs protected by an N_2 atmosphere. The reaction was allowed to cool and the solvent removed under reduced pressure, the resulting purple solid was dissolved in EtOAc (50ml) and washed with 2M NaOH(aq) (3x50ml), brine (2x50ml) and dried with Na_2SO_4. Solvent was removed under reduced pressure and the crude product purified on silica (100g). Fractions containing the desired product were condensed under reduced pressure to give 2 (0.43g, 0.36 mmol, 46%) of purple solid.

^{1}H NMR (400MHz, d-CDCl3) δ 8.92 (s, 2H), 7.83 (d, 1H, J=4 Hz), 7.79 (s, 1H), 7.66, (dd, 1H, J=8.1Hz, J=8 Hz), 7.34 (d, 1H, J=8 Hz), 4.20 (t, 2H, J=6 Hz), 4.14 (t, 2H, J=8), 2.44 (t, 2H, J=8 Hz) 1.97-1.90 (m, 4H,), 1.25 (t, 3H, J=7 Hz), -2.79 (s, 0.5H).

FTIR (neat, ν_{max} cm^{-1}) 3300, 2930, 2880, 1729, 1594 ;

MS (ES+) m/z (%) = 596.3 [M+2H]2+ (30), 1191.5 [M+H]1+ (100) ;

HRMS (ES+): calculated for $C_{72}H_{79}N_4O_{12}$: 1191.5695 ; found 1191.5694 ;

UV/Vis (DMSO) λ_{max}/nm (ε/mol^{-1}cm^{-1}) 421 (ε=3.8*10^5), 514 (ε=2.0*10^4), 549 (8.0*10^3), 590 (ε=6.6*10^3), 644 (ε=4.9*10^3).

5,10,15,20-Tetrakis(ethyl-6-(3-phenoxy)hexanoate)-21H,23H-porphyrin, 3

To 1 (1.283g, 1.89 mmol) and Cs2CO3 (2.96g, 9.07 mmol), acetonitrile (Dry)(100ml) was added. The mixture was refluxed for 15 mins while protected by an N_2 atmosphere. Then ethyl 6-bromohexanoate (2.02g, 1.61ml, 9.07 mmol) was added and left to reflux protected by an N_2 atmosphere for 18 hrs. The reaction vessel was allowed to cool to room temperature and the solvent was removed under reduced pressure. The resulting purple gum was dissolved in EtOAc (100ml) and washed with 2M NaOH (3x100ml), brine (2x100ml) and dried with Na2SO4. Solvent was removed under reduced pressure, the crude product purified on silica (100g) eluting with DCM:EtOAc. Fractions containing only the desired product (by TLC) were condensed under reduced pressure to give 3 (0.856g, 0.69 mmol, 36%) of purple solid.

^{1}H NMR (400MHz, d-$CDCl_3$) 8.81 (s, 2H), 7.71 (d, 1H, J=8), 7.68 (s, 1H), 7.54 (dd, 1H, J=8 , J=8), 7.22 (d, 1H, J=8), 4.07-3.99 (m, 4H), 2.25 (t, 2H, J=7 , 1.83-1.77 (m, 2H) 1.66-1.60 (m, 2H), 1.52-1.44 (m, 2H),1.13 (t, 3H, J=8) -2.95 (s, 0.5H);

FTIR (neat, ν_{max} cm^{-1}) 3316, 2934, 2866, 1728, 1594, 1574;

MS (ES+) m/z (%) = 624.3 [M+2H+] (25), 1247.7 [M+H+] (100);

HRMS (ES+): calculated for $C_{76}H_{87}N_4O_{12}$: 1247.6320 ; found 1247.6307;

UV/Vis (DMSO) λ_{max}/nm (ε/mol^{-1}cm^{-1}) 422 (ε=3.2*10^5), 514 (ε=6.7*10^3), 550 (ε=2.4*10^3), 588 (ε=1.9*10^3), 644 (ε=1.3*10^3).

5,10,15,20-Tetrakis(ethyl-7-(3-phenoxy)heptanoate)-21H,23H-porphyrin, 4

To 1 (0.51 g, 0.75 mmol) and Cs_2CO_3 (1.18 g, 3.61 mmol) was added acetonitrile (50ml). The reaction was stirred while protected by an N_2 atmosphere for 15 mins at reflux. Ethyl-7-bromoheptanoate (0.86g, 0.70ml, 3.61 mmol) was added and the reaction mixture was refluxed for 41 hrs protected by a N_2 atmosphere. The reaction mixture was allowed to cool and the solvent was removed under reduced pressure. The product was dissolved in EtOAc (50ml) and washed with 2M NaOH (3x50ml), brine (2x50ml) and dried with Na2SO4. Solvent was removed under reduced pressure and the crude product purified on silica (100g). Fractions containing only the desired product were condensed under reduced pressure to give 4 (0.53g, 0.41 mmol, 54%) of purple solid.

^{1}H NMR (400MHz, d-$CDCl_3$) δ 8.91 (s, 2H), 7.82 (d, 1H, J = 8), 7.79 (s, 1H), 7.65 (dd, 1H, J = 8, J = 8), 7.34 (d, 1H, J = 7.8), 4.16 (t, 2H, J = 6.), 4.12 (q, 2H, J =7), 2.34-2.30 (m, 2H), 1.94-1.87 (m, 2H, 1.73-1.65 (m), 1.61-1.53 (m, 2H), 1.48-1.42 (m, 2H), 1.24 (t, 3H, J=7,), -2.79 (s, 0.5H),

^{13}C NMR (100MHz, d-$CDCl_3$); 173.8, 157.5, 143.4, 127.6, 127.5, 121.0, 119.9, 114.1, 68.1, 60.2, 34.3, 29.3, 28.9, 25.8, 24.9, 14.24.

FTIR (neat, vmax cm-1) 3300, 2930, 2850, 1731, 1595;

MS (ES+) m/z (%)= 652 (40), 1304 [M+H+] (100);

HRMS (ES+): calculated for $C_{80}H_{95}N_4O_{12}$: 1304.65 ; found 1303.6946;

UV/Vis (DMSO) λ_{max}/nm (ε/mol^{-1}cm^{-1}) 421 (ε=3.9*10^5), 514 (ε=1.7*10^4), 549 (ε=6.2*10^3), 588 (ε=5.1*10^3), 646 (ε=3.5*10^3)

5,10,15,20-Tetrakis(5-(3-phenoxy)pentanoic acid)-21H,23H-porphyrin, C5SHU

To 3 (0.32g, 0.27 mmol), methanol (50ml) was added, brought to reflux then allowed to cool. Potassium hydroxide (0.60g, 10.74 mmol) was added to the solution, brought to reflux and left overnight. The solvent was removed under reduced pressure and dissolved in deionised water (250ml), washed with DCM (150ml) and neutralised with 5% citric acid (200ml). The solution was checked with indicator paper and the product extracted with DCM (3x80ml). The organic layer was collected and condensed under reduced pressure to give 5 (0.20g, 0.19 mmol, 69%) of purple solid.

^{1}H NMR (400MHz, d_6-DMSO) 12. 05 (s, 1H), 8.89 (s, 2H), 7.79-7.77 (m, 2H), 7.69 (dd, 1H, J=8 Hz, J=8 Hz), 7.40 (d, 1H, J=8 Hz), 4.17 (t, 2H, J=6 Hz), 2.30 (t, 2H, J=7 Hz), 1.83-1.68 (m, 4H) -2.94 (s, 0.5H);

FTIR (neat, ν_{max} cm^{-1}) 3350, 2940, 2890, 1704, 1595;

MS (ES+) m/z (%)= 1080 [M+H+](100);

HRMS (ES+): calculated for $C_{64}H_{62}N_4O_{12}$: 1079.4442 ; found 1079.4417;

UV/Vis (DMSO) λ_{max}/nm (ε/mol-1cm-1) 421 (ε=2.2*105), 515 (ε=1.0*104), 550 (ε=4.1x103), 590 (ε=3.3 x103), 647 (ε=2.3 x103).

5,10,15,20-Tetrakis(6-(3-phenoxy)hexanoic acid)-21H,23H-porphyrin, C6SHU

To a suspension 3 (0.096g, 0.077 mmol) in methanol (25ml) was added potassium hydroxide (0.34g, 6.2 mmol) brought to reflux and left overnight. The solvent was removed under reduced pressure and dissolved in deionised water (50ml), washed with DCM (50ml) and neutralised with 5% citric acid. The aqueous layer was checked with indicator paper and the product extracted with DCM (2x50ml). The organic layer was collected and condensed under reduced pressure to give 6 (0.086g, 0.075 mmol, 98%) of purple solid.

^{1}H NMR (400MHz, d_6-DMSO) δ: 8.87 (s, 2H), 7.77 (m, 2H), 7.69 (dd, 1H, J=8Hz, J=8Hz), 7.39 (d, 1H, J=8Hz), 4.16 (t, 2H, J=6Hz), 2.23-2.19 (m, 2H), 1.83-1.77 (m, 2H), 1.59-1.53 (m,2H), 1.49-1.44 (m, 2H), -2.95 (s, 0.5H);

FTIR (neat, ν_{max} cm^{-1}) 2918, 2861, 1702.43, 1592, 1573;

MS (ES-) m/z (%)=, 566.3 [M-2H] (45), 1133.5 [M-H](100);

HRMS (ES-): calculated for $C_{68}H_{69}N_4O_{12}$: 1133.4917; found 1133.4915;

UV/Vis (DMSO) λmax/nm (ε/mol^{-1}cm^{-1}) 421 (ε=1.9*10^5), 515(ε=7.3*10^3), 550(ε=2.7x10^3), 590(ε=2.2 x10^3), 647(ε=1.6 x10^3).

5,10,15,20-Tetrakis(7-(3-phenoxy)heptanoic acid)-21H,23H-porphyrin, C7SHU

To 4 (0.50g, 0.84 mmol), methanol (50ml) was added, brought to reflux then allowed to cool. Potassium hydroxide (0.86g, 15.3 mmol) was added to the solution, brought to reflux and left overnight. The solvent was removed under reduced pressure and dissolved in deionised water (250ml), washed with DCM (150ml) and neutralised with 5% citric acid (200ml). The solution was checked with indicator paper and the product extracted with DCM (3x80ml). The organic layer was collected and condensed under reduced pressure to give 6 (0.43g, 0.36 mmol, 94%) of purple solid.

^{1}H NMR (400MHz, d6-DMSO) 8.87 (s, 2H), 7.76-7.74 (m, 2H), , 7.68 (dd, 1H, J=8 Hz, J = 8 Hz), 7.39 (d, 1H, J=8 Hz), 4.14 (t, 2H, J=6 Hz), 2.16 (m, 2H), 1.79-1.76 (m, 2H), 1.50-1.41 (m, 4H), 1.36-1.24 (m, 2H), -2.96 (s, 0H) ;

FTIR (neat, ν_{max} cm^{-1}) 3300, 2931, 2880, 1702, 1594;

MS (ES+) m/z (%)= 1191.6 [M+H+] (100);

HRMS (ES+): calculated for $C_{72}H_{79}N_4O_{12}$: 1191.5728 [M+H+] ; found 1191.5729;

UV/Vis (DMSO) λ_{max}/nm (ε/mol^{-1}cm^{-1}) 421 (ε=5*10^5), 515 (ε=2.2*10^4), 550 (ε=8.2x10^3), 590 (ε=6.6x10^3), 645 (ε=4.7x10^3).

NMR Spectra

5,10,15,20-Tetrakis(ethyl-5-(3-phenoxy)pentanoate)-21H,23H-porphyrin, 2

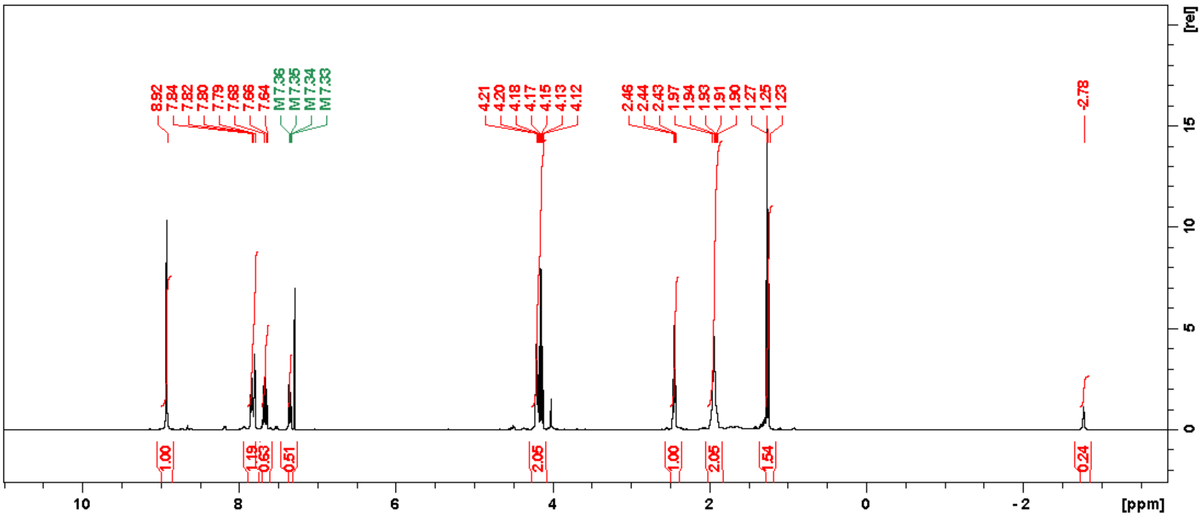

5,10,15,20-Tetrakis(ethyl-6-(3-phenoxy)hexanoate)-21H,23H-porphyrin, 3

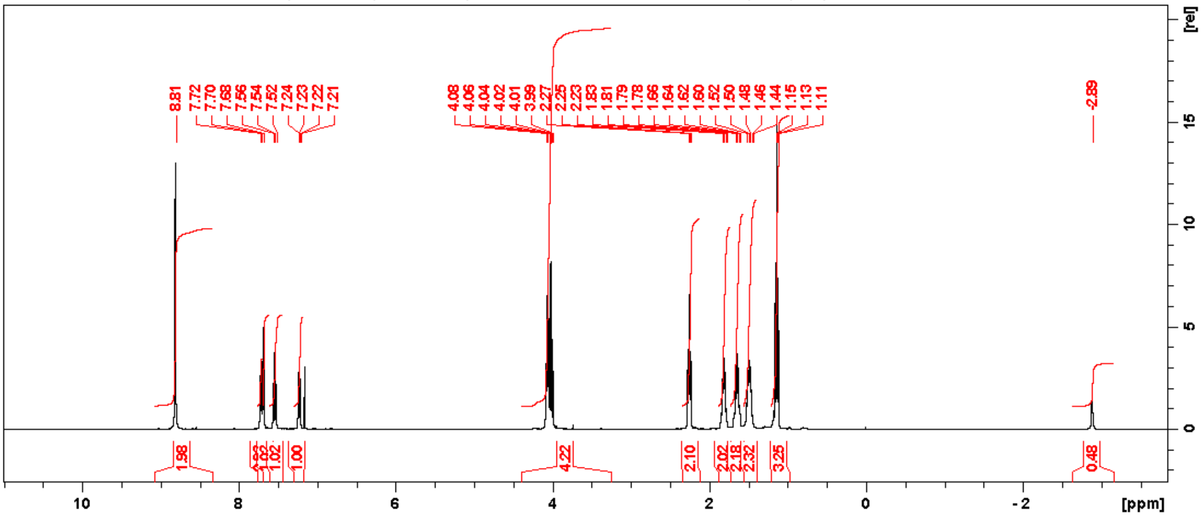

5,10,15,20-Tetrakis(ethyl-7-(3-phenoxy)heptanoate)-21H,23H-porphyrin, 4

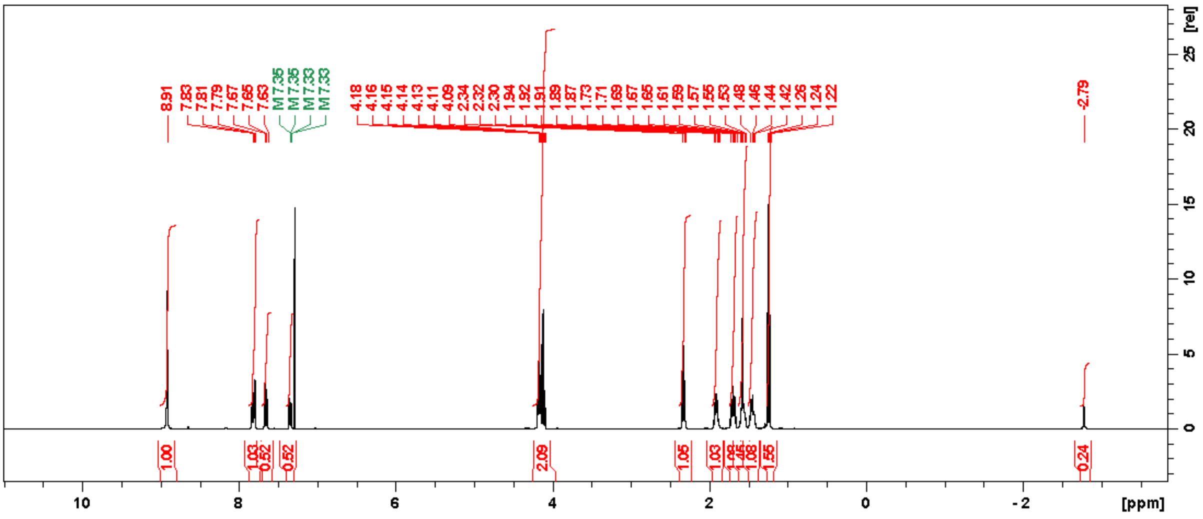

5,10,15,20-Tetrakis(5-(3-phenoxy)pentanoic acid)-21H,23H-porphyrin, C5SHU

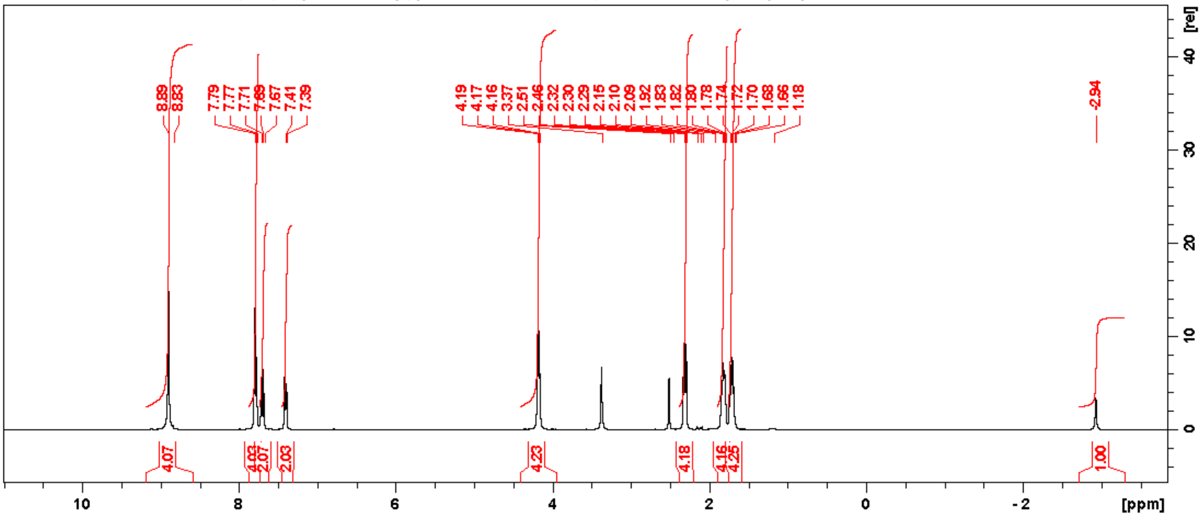

5,10,15,20-Tetrakis(6-(3-phenoxy)hexanoic acid)-21H,23H-porphyrin, C6SHU

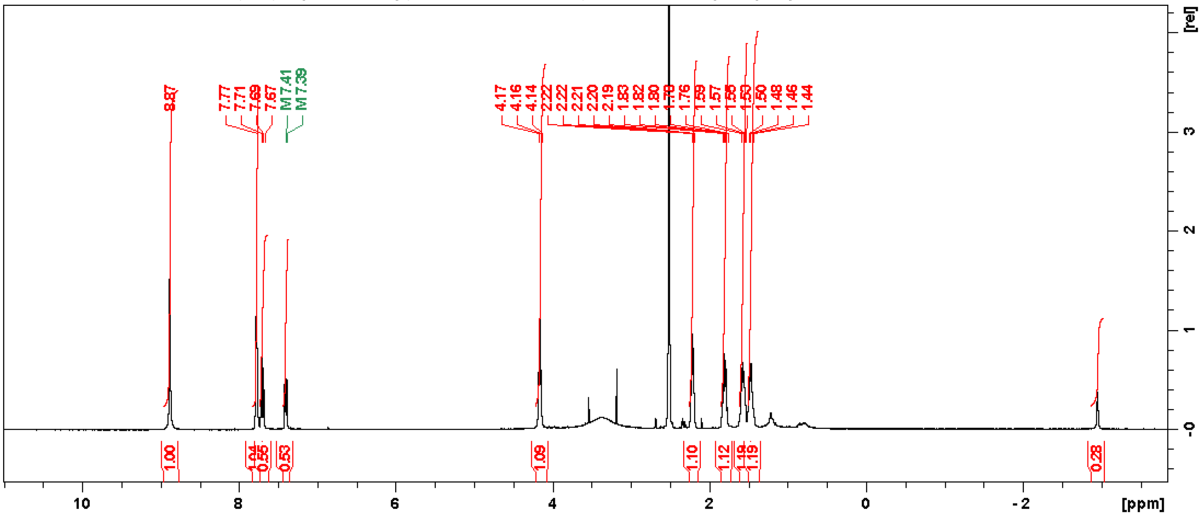

5,10,15,20-Tetrakis(7-(3-phenoxy)heptanoic acid)-21H,23H-porphyrin, C7SHU

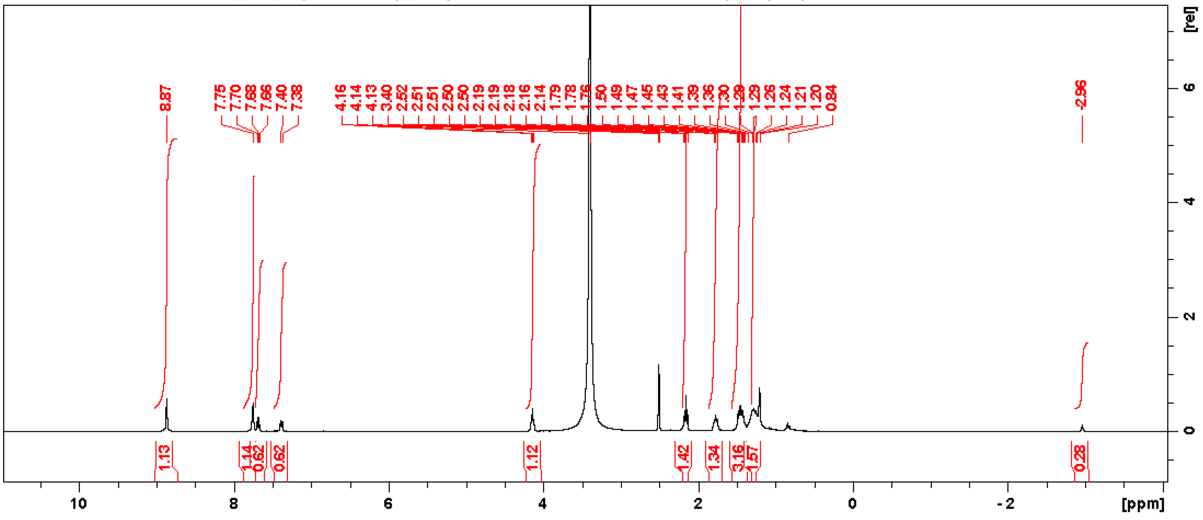

Mass spectrometry data for C5SHU, C6SHU and C7SHU

Mass spectrometry for C5SHU

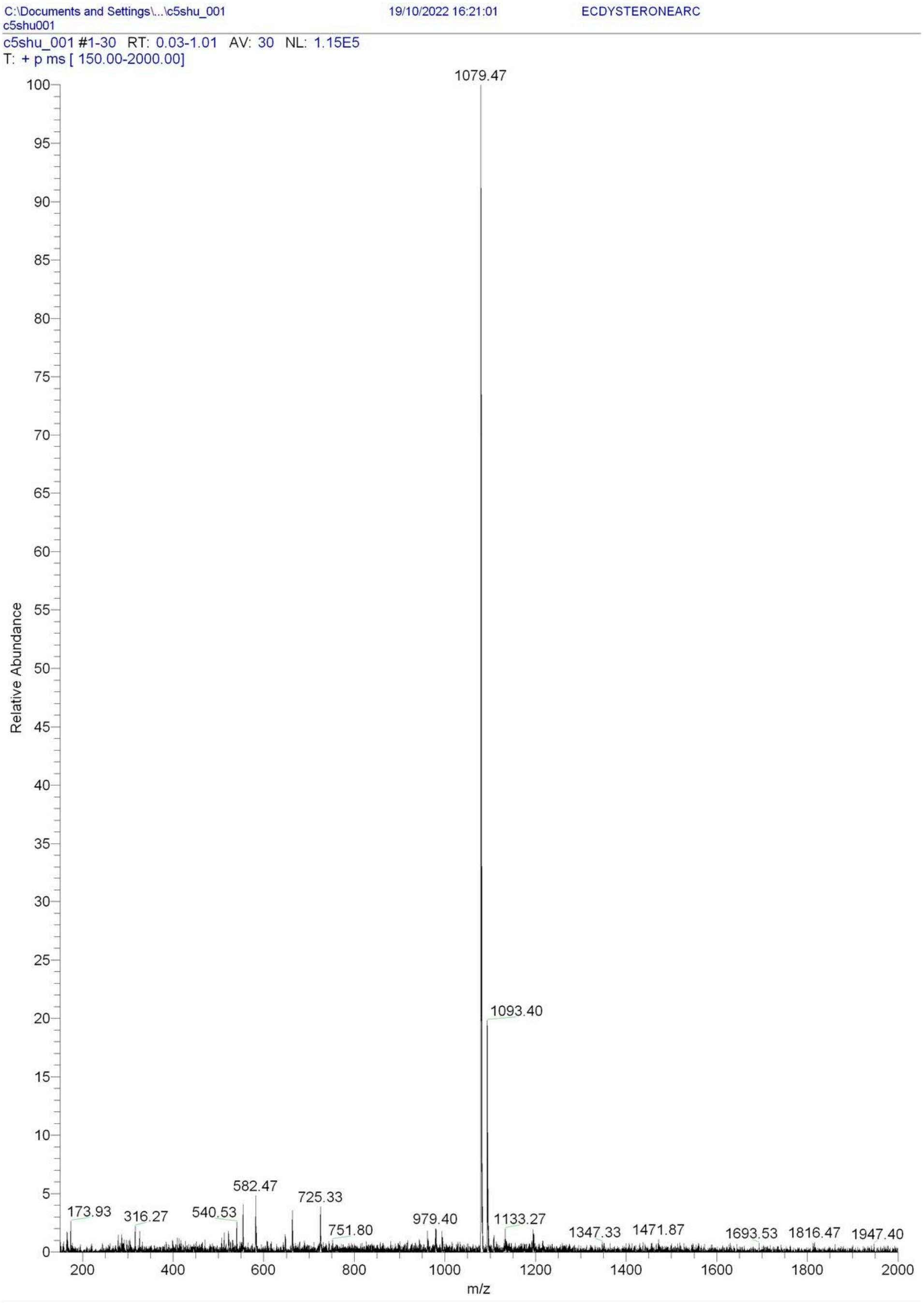

Mass spectrometry for C6SHU

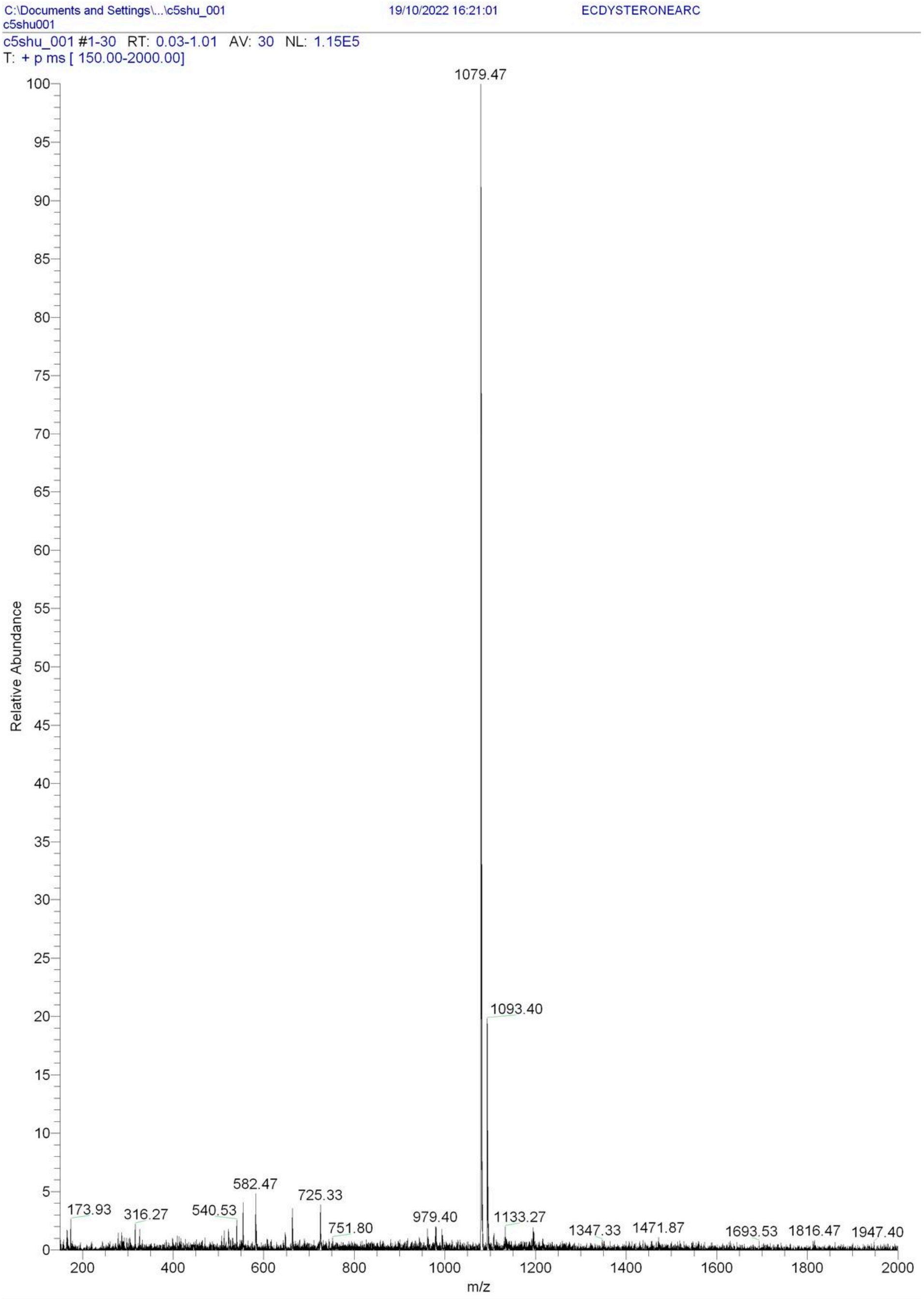

Mass spectrometry data for C7SHU

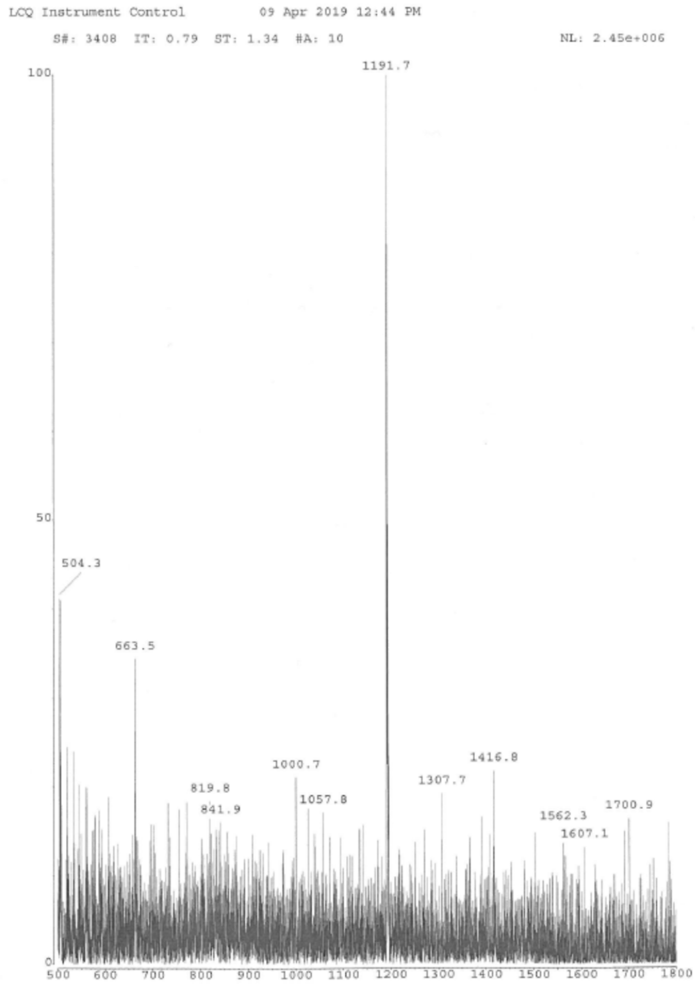

Representative UV/vis absorbance coefficient measurement in DMSO

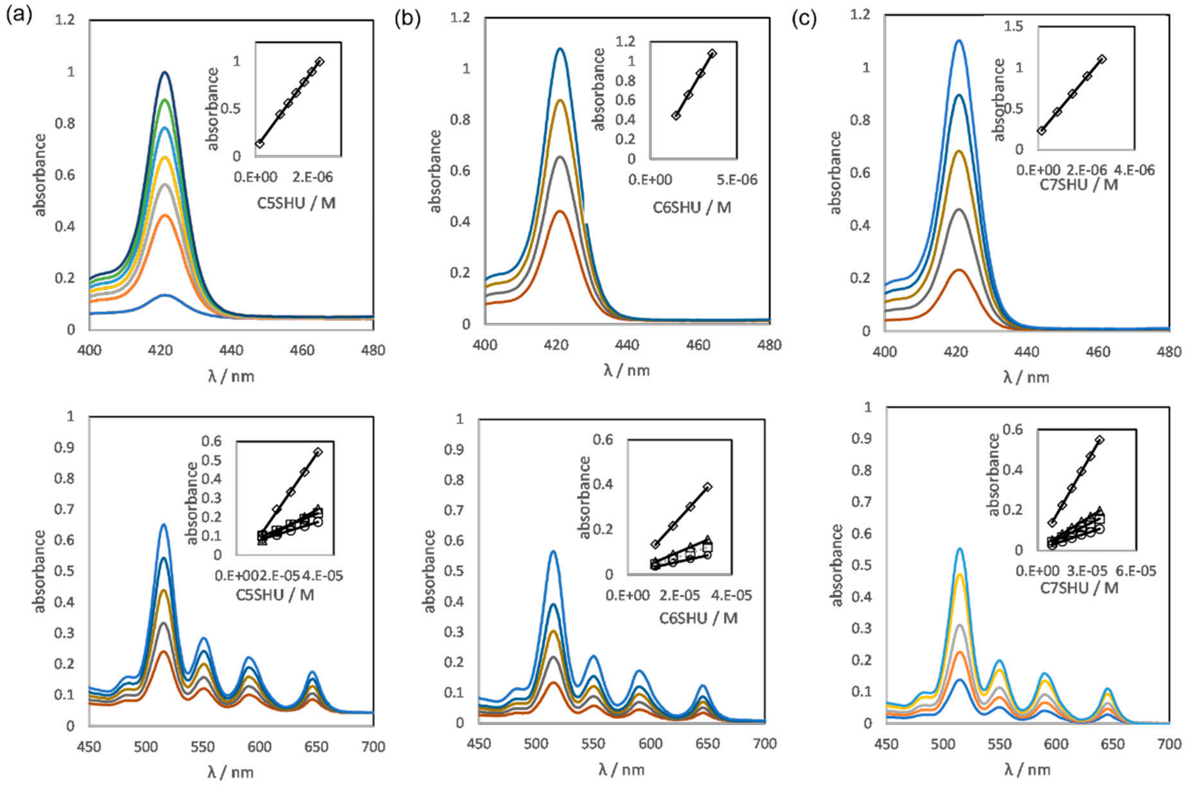

Figure S1. UV/vis absorbance coefficient measurement in DMSO for(a) C5SHU Soret band 421 nm R^2 = 1.000, Q-band 515 nm R^2 = 0.998, Q-band 550 nm R^2 = 0.999, Q-band 590 R^2 = 0.998 nm and Q-band 647 nm R^2 = 0.998 (b) C6SHU Soret band 421 nm R^2 = 1.000, Q-band 515 nm R^2 = 0.999, Q-band 550 nm R^2 = 0.999, Q-band 590 R^2 = 0.999 nm and Q-band 647 nm R^2 = 0.999 (c) C7SHU Soret band 421 nm R^2 = 1.000, Q-band 515 nm R^2 = 1.000, Q-band 550 nm R^2 = 0.999, Q-band 590 R^2 = 0.999 nm and Q-band 645 nm R^2 = 0.997.

MSTO-211H + C5SHU 24-hour treatment

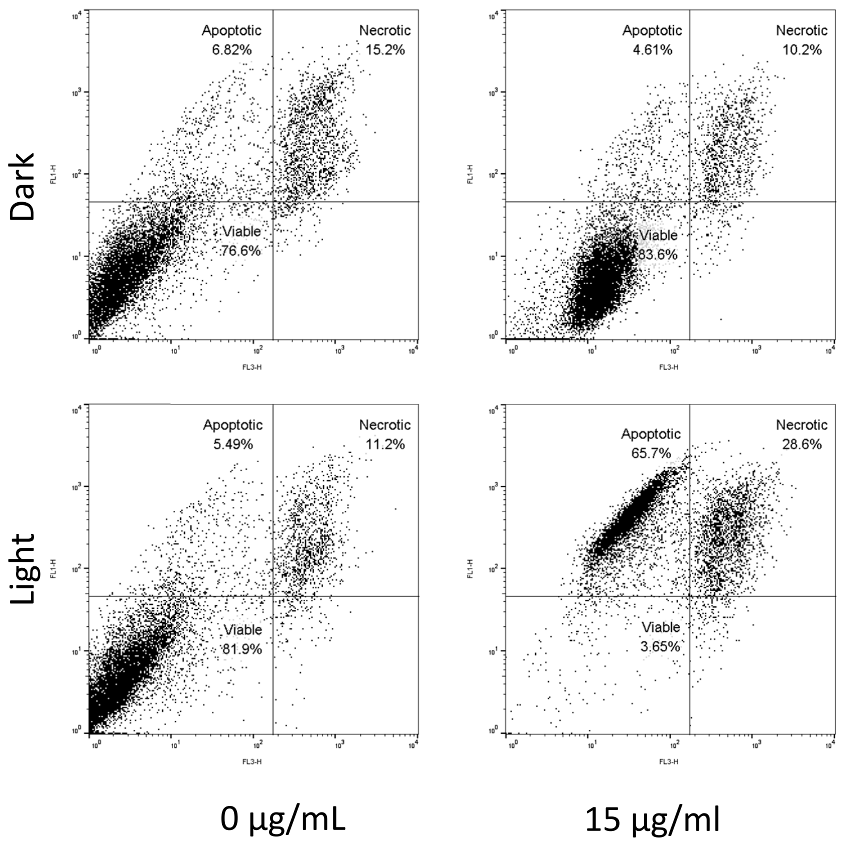

MET-5a + C5SHU 24-hour treatment

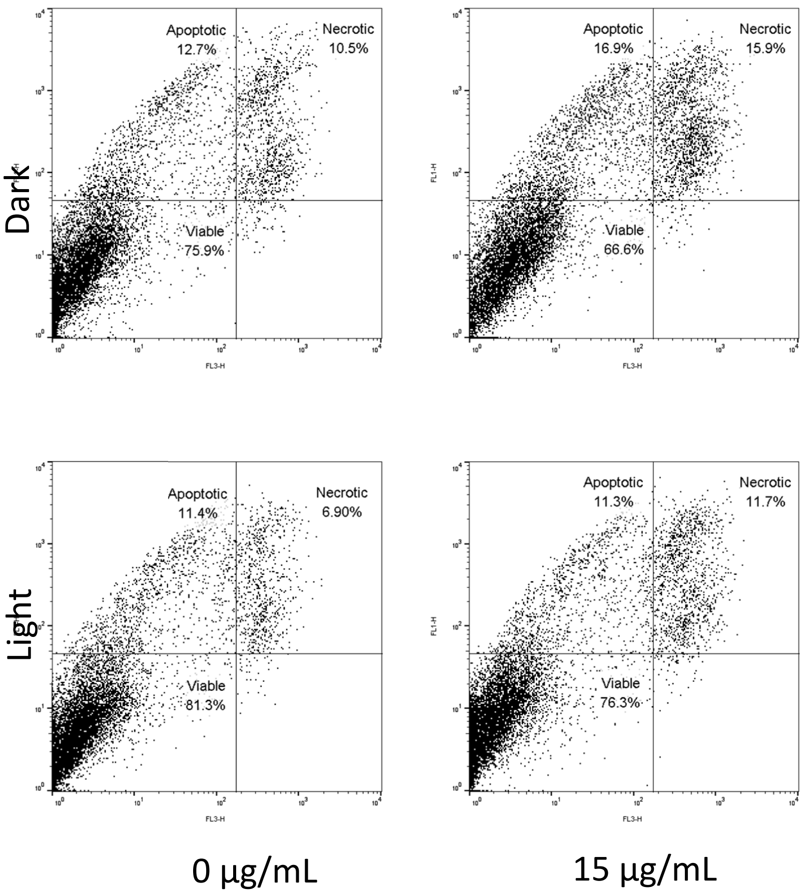

H28 + C5SHU 24-hour treatment

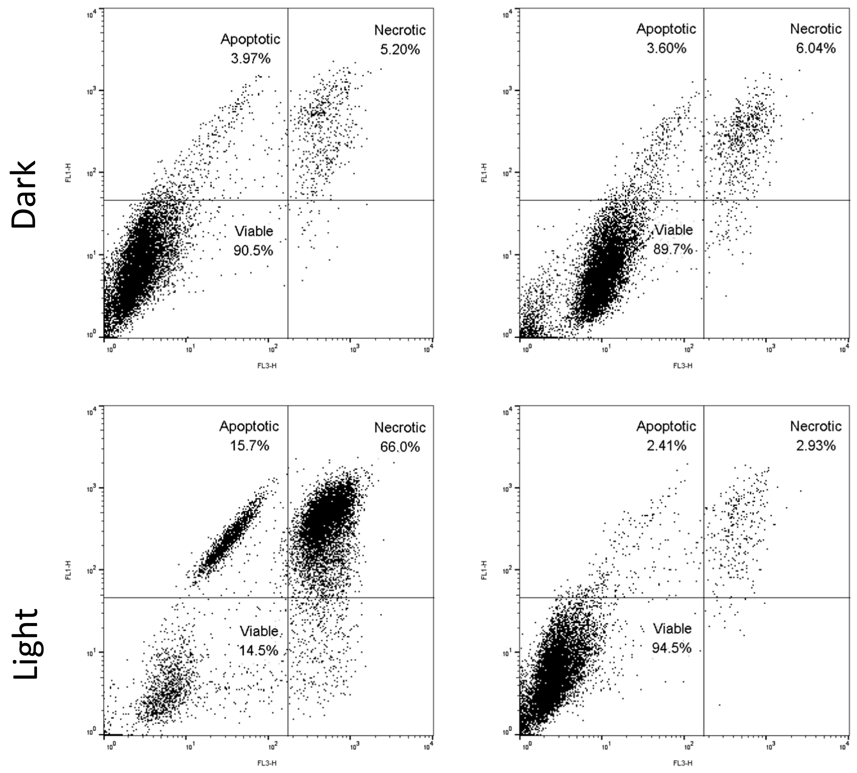

0 µg/mL 15 µg/mL

Figure S2. Representative C5SHU-treated MSTO-211H, NCI-H28 and Met-5a cells as determined by the Annexin V-FITC/PI assay 24 hours after treatment. Each density plot shows viable cells (Annexin⁻ PI⁻), apoptotic cells (Annexin⁺ PI⁻); and necrotic cells (Annexin⁻ PI⁺).

Funding
The authors are grateful to acknowledge funding provided by Sheffield Hallam University.

Author Contributions
Sam Bonsall, Simeon Hubbard (data curation and investigation, original draft preparation), Uthaman Jithin, Joseph Anslow, Dylan Todd, Callum Rowding, Tom Filarowski, Greg Duly, Ryan Wilson, Jack Porter (data curation and investigation) Simon Turega & Sarah Haywood-Small (conceptualization; methodology, reviewing and editing and project supervision).

Institutional Review Board Statement
Not applicable

Conflicts of Interest
The authors declare no conflict of interest
